# New Research in Food Allergen Detection

**DOI:** 10.3390/foods11101520

**Published:** 2022-05-23

**Authors:** Rosario Linacero, Carmen Cuadrado

**Affiliations:** 1 Genetics, Physiology and Microbiology Department, Biology Faculty, Complutense University, 28040 Madrid, Spain; 2 Food Technology Department, Instituto Nacional de Investigación Agraria y Alimentaria-CSIC, Ctra. La Coruña Km. 7.5, 28040 Madrid, Spain; cuadrado@inia.csic.es

Food allergy is a worldwide health problem that concerns all ages from infants to adults. The presence of undeclared allergenic ingredients or the presence of traces of allergens due to contamination during food processing poses a great health risk to sensitized individuals. Therefore, reliable analytical methods are required to detect and identify allergenic ingredients in food products. The enzyme-linked immunosorbent assay (ELISA) is the most used method to detect small amounts of proteins from specific foods, and it is possible to find several ELISA kits, as well as other commercial immunoassays (i.e., lateral flow), in the market. In recent years, DNA-based methodologies have been proposed as a specific, sensitive and reliable alternative to ELISA, such as real-time PCR, microarrays and DNA biosensors. 

The present Special Issue “New Research in Food Allergen Detection” provides an updated overview of the applications of new research in DNA- and protein-based methodologies for the detection of allergens. Real-time PCR has been used to develop new, specific and efficient primer and probe systems for the 2S albumin gene for sesame and pistachio and for the vicilin precursor gene for macadamia nut [1]. These systems were subjected to a robust intra-laboratory qualitative validation process prior to their application, by DNA extraction and fast real-time PCR, on some real market samples to reproduce a potential allergen contamination along the food chain. The developed system results were specific and robust, with a sensible limit of detection (0.005% for sesame; 0.004% for pistachio; 0.006% for macadamia nut). The performance and the reliability of the sesame, pistachio and macadamia target systems were confirmed on commercial food samples.

For the detection of peanut allergens by real-time PCR, three chloroplast markers (matK, rpl16 and trnH-psbA) and TaqMan probes were used by Sanchiz et al. [2] for the specific detection of peanut, in order to increase the assay sensitivity. Efficiency and linear correlation of calibration curves were within the adequate ranges. The MatK chloroplast marker yielded the most sensitive and efficient detection for peanut. Moreover, the detection of matK in binary mixtures of peanut processed samples was possible for up to 10 mg/kg, even after boiling, and autoclaving at 121 °C for 15 min, with acceptable efficiency and linear correlation. The applicability of this method was assayed in several commercial food products. The effect of instant controlled pressure drop (DIC) treatment on the detection of nut allergens was determined by real-time PCR. According to Vicente et al. [3], the detection of targets in hazelnut, pistachio and cashew (Cor a 9, Pis v 1 and Ana o 1, respectively) is affected by the treatment to different extents depending on the tree nut. The results were compared to those previously obtained in the analysis of other treatments (autoclave) on the amplificability of the same targets. The reduction in amplificability was similar to that reported for some autoclave conditions. These assays might allow for the detection of up to 1000 mg/kg of hazelnut, pistachio and cashew flours after being submitted to DIC treatment in food matrices.

Soybean detection in complex food matrices by loop-mediated isothermal amplification (LAMP) of the multicopy gene ORF160b, combined with a lateral flow dipstick (LFD)-like detection, was developed [4]. The results were compared with those obtained using quantitative real-time polymerase chain reaction (qPCR) as the current standard of DNA-based allergen detection, and an antibody-based commercial lateral flow device (LFD) as the current reference of protein-based rapid allergen detection. LAMP-LFD allowed unequivocal and reproducible detection of 10 mg/kg soybean in three different matrices (boiled sausage, chocolate, instant tomato soup). Therefore, the sensitivity of soybean detection in food matrices, commercial retail samples and various processed soybean products was comparable between LAMP-LFD and qPCR. The DNA-based LAMP-LFD proved to be a simple and low-technology soybean detection tool, showing sensitivity and specificity that are comparable or superior to the investigated commercial protein-based LFD.

Celiac disease and other gluten-related diseases are presenting increasing prevalences, for which a strict gluten-free diet is the best treatment. Due to this situation, gluten labeling legislation has been developed in several countries around the world. Several immunological-based techniques for gluten detection in food samples have been applied to comply with such regulations. According to García-Calvo et al. [5], the strategies used for developing these antibodies can be summarized as follows: (1) polyclonal antibodies raised by animal immunization comprised the first method that is still currently in use, with two main variants: mammal-derived IgG and chicken-derived IgY; (2) monoclonal antibodies such as 401.21 and R5 were produced from hybridomas raised against different cereal protein extracts; (3) monoclonal antibodies such as G12, produced from hybridomas, were raised against recombinant proteins that were implied in celiac pathogenesis; and (4) recombinant antibodies were obtained by directed molecular evolution with phage or cDNA display technology. Each of these technologies outlined (polyclonal, monoclonal and recombinant antibodies) has advantages and disadvantages. The selection of the appropriate methodology will depend on the intended use and resources available. Initially, the main objective was the consecution of new high-affinity antibodies, resulting in low detection and quantification limits that are mainly achieved with the R5 mAb (the gold standard for gluten detection). Increasing knowledge about the causes of gluten-related diseases has increased the complexity of research in this field, with current efforts focusing not only on the development of more specific and sensitive systems for gluten but also on the detection of protein motifs related to pathogenicity. New tools based on recombinant antibodies will provide adequate safety and traceability methodologies to meet the increasing market demand for gluten-free products. 

Phage displayed domain antibodies (dAb) for the detection of allergenic pistachio proteins in foods were developed by Madrid et al. [6]. Several phage display biopanning strategies were evaluated to screen the human-based domain antibody library (dAb) in search for pistachio-specific probes. The clone producing the PVF4 phage-dAb was finally selected, and it did not cross-react with cashew despite the phylogenetic proximity with pistachio. Western blot and matrix-assisted laser desorption/ionization tandem mass spectrometry (MALDI-TOF/TOF) analysis demonstrated that this clone recognized a unique band of ∼22 kDa related to the basic subunit of pistachio 11S globulin (allergen Pis v 2). The PVF4 phage-dAb allowed the detection of pistachio in a food matrix with a limit of detection (LOD) of 3983 mg kg^−1^ in an indirect phage-enzyme-linked immunosorbent assay (ELISA). The ELISA method developed was used to assess the applicability of the PVF4 phage-dAb for analysis of 77 commercial food products.

Seafood is considered one of the main food allergen sources by the European Food Safety Authority (EFSA). It comprises several distinct groups of edible aquatic animals, including fish and shellfish, such as crustaceans and mollusks. Recently, the EFSA recognized the high risk of food allergy over the world and established the necessity of developing new methodologies for its control. Consequently, accurate, sensitive and fast detection methods for seafood allergy control and detection in food products are highly recommended. Proteomics-based methodologies for the detection and quantification of seafood allergens are useful and highly recommended and were reviewed by Carrera et al. [7]. For this purpose, two consecutive proteomics strategies (discovery and targeted proteomics) are applied to the study and control of seafood allergies. In the discovery proteomics approach, a mixture of proteins is separated using two-dimensional gel electrophoresis (2-DE), the spots of interest are in-gel digested with trypsin, the peptides obtained are analyzed using liquid chromatography coupled to tandem mass spectrometry (LC-MS/MS) and the spectra are identified using different database search engines, such as SEQUEST and PEAKS. Specific peptide biomarkers can be selected. In the fast targeted proteomics approach, the mixture of proteins is in-solution digested with trypsin and accelerated using high-intensity focused ultrasound (HIFU), and then the peptide biomarkers selected in the discovery approach can be monitored using selected MS/MS ion monitoring or parallel reaction monitoring (PRM) during mass spectrometry. The monitoring of peptide biomarkers can be performed in less than 2 h. In addition, future directions and new perspectives are also provided. New mass spectrometer modes for data-independent acquisition (DIA), such as LC-MSE or SWATH, combined with high-resolution mass spectrometers (HRMSs) will largely improve the detection and quantification of traces of seafood allergens in different foodstuffs. In addition, DIA coupled with ion mobility mass spectrometry (DIA-IM-MS) will be relevant to investigating the allergen composition in a challenge mixture of ingredient meals. Furthermore, the application of absolute quantitation using AQUA-LC-MRM, the use of capillary electrophoresis (CE) coupled with a top-down proteomics approach to detect intact protein allergens in HRMS instruments and the employment of new complementary top-down MS/MS fragmentation modes (high-energy collisional dissociation (HCD), electron-transfer/high-energy-collisional dissociation (ETDhcD) and ultraviolet photodissociation (UVPD)) for the characterization and de novo sequencing of whole allergens are new directions that will provide new valuable insights.

The increasing development of edible insect flours as alternative sources of proteins added to food and feed products for improving their nutritional value necessitates an accurate evaluation of their possible adverse side effects, especially for individuals suffering from food allergies. According to Barre et al. [8], a proteomics- and bioinformatics-based approach can be used for the identification of specific allergens from edible insects such as silkworm (*Bombyx mori*), cricket (*Acheta domesticus*), African migratory locust (*Locusta migratoria*), yellow mealworm (*Tenebrio molitor*), red palm weevil (Rhynchophorus ferrugineus) and giant milworm beetle (*Zophobas atratus*). Most of them consist of phylogenetically related protein allergens widely distributed in the different groups of arthropods (mites, insects, crustaceans) and mollusks. However, a few proteins belonging to discrete protein families including chemosensory proteins, hexamerins and odorant-binding proteins emerged as proteins highly specific for edible insects. To a lesser extent, other proteins such as apolipophorin III, the larval cuticle protein and the receptor for activated protein kinase also exhibited a rather good specificity for edible insects. These proteins, which are apparently missing or much less represented in other groups of arthropods, mollusks and nematodes, share well conserved amino acid sequences and very similar three-dimensional structures. Owing to their ability to trigger allergic responses in sensitized people, they should be used as probes for the specific detection of insect proteins as food ingredients in various food products and thus to assess their food safety, especially for people allergic to edible insects.
